# Phase 1 clinical trial evaluating safety, bioavailability, and gut microbiome with a combination of curcumin and ursolic acid in lipid enhanced capsules

**DOI:** 10.1016/j.jtcme.2024.03.002

**Published:** 2024-03-07

**Authors:** Michael A. Liss, Furkan Dursun, G. Lavender Hackman, Mohamed I. Gadallah, Achinto Saha, Chelsea A. Friedman, Atul S. Rathore, Preeti Chandra, James R. White, Stefano Tiziani, John DiGiovanni

**Affiliations:** a Department of Urology, University of Texas Health San Antonio, San Antonio, TX, USA; b Department of Nutritional Sciences, College of Natural Science, The University of Texas at Austin, USA; c Department of Pharmaceutical Analytical Chemistry, Faculty of Pharmacy, Assiut University, Assiut, 71526, Egypt; d Division of Pharmacology & Toxicology, College of Pharmacy, The University of Texas at Austin, Austin, TX, USA; e Department of Pediatrics, Dell Medical School, The University of Texas at Austin, Austin, TX, 78723, USA; f Department of Oncology, Dell Medical School, Livestrong Cancer Institutes, The University of Texas at Austin, Austin, TX, 78723, USA; g Resphera Biosciences, USA; h Center for Molecular Carcinogenesis and Toxicology, The University of Texas at Austin, Austin, TX, USA

**Keywords:** Phytochemicals, Prostate, Cancer prevention, Microbiome, Ursolic acid, Curcumin

## Abstract

As screening strategies employ better biomarkers and genetics to identify individuals at an increased risk of prostate cancer, there are currently no chemotherapeutic prevention strategies. With any chemoprevention strategy, the population will be younger and healthier; therefore, they will be less tolerant of side effects. This study translated findings from screening a natural product library and pre-clinical evaluation of curcumin (CURC) in combination with ursolic acid (UA) in prostate cancer models. After manufacturing capsules for each compound, 18 subjects were enrolled. The study used a 3 × 3 phase 1 clinical trial to evaluate CURC (1200 mg/day) and UA (300 mg/day) alone and in combination over a 2-week period with endpoints of safety, bioavailability, and microbiome alterations. After enrolling six subjects in each arm, we found no grade 3 or 4 events and only minor changes in the safety laboratory values. In the pooled analysis of groups, we noted a statistically significant difference between median serum levels of UA when administered alone vs administered in the combination (2.7 ng/mL vs 43.8 ng/mL, p = 0.03). Individuals receiving the combination also had a favorable impact on gut microbiome status and a reduction in “microbiome score” predictive of prostate cancer risk.

## Graphical abstract

**Figure:**
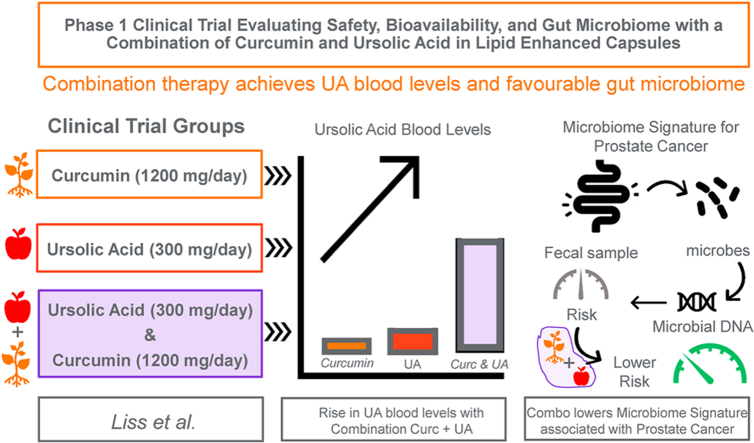

## Abbreviations

AGC –: Automatic gain control
ALT –: Alanine aminotransferase
CTCAE -: Common Terminology Criteria for Adverse Events
CURC –: Curcumin
CurcUA –: Curcumin and Urosolic Acid
DNA –: Deoxyribonucleic Acid
DSMB –: Data Safety Monitoring board
GMP –: good manufacturing practice
IRB –: Internal Review Board
IT –: injection time
LC: Liquid chromatography
MDR1: Multidrug resistant 1 gene
OUT –: operational taxonomic unit
PC-3 –: Prostate Cancer 3 mouse model
PCa –: Prostate Cancer
PICRUSt –: Phylogenetic Investigation of Communities by Reconstruction of Unobserved States
PRM –: Parallel reaction monitoring
QIIME -: Quantitative Insights Into Microbial Ecology
RNA –: Ribonucleic acid
SABOR –: San Antonio Biomarkers of Risk
SwRI –: Southwest Research Institute
UHPLC –: Ultra High-Performance liquid chromatography
UA: Ursolic Acid
V1–V2 –: Variable region 1 and 2 of 16s rRNA
V3–V4 –: Variable region 3 and 4 of 16s rRNA

## 1 Introduction

As new biomarkers identify men at increased risk for prostate cancer (PCa), the need for risk reduction strategies, such as chemoprevention, is likely to increase. The long latency period of carcinogenesis makes prostate cancer an ideal target for chemoprevention. The average age for diagnosis of PCa is 66 years^1^; however, the onset of pre-clinical disease may occur in adults as early as 30 years of age.^2^ This long lag time provides an ideal opportunity to target prostate cancer by minimizing the progression of precancerous lesions. Because the cancer prevention target group is largely young and healthy, the ideal therapy would be very safe with few side effects. In addition, an estimated 1.5 million men have prostate cancer (PCa). If low-grade PCa is detected, it is usually monitored, which also provides an opportunity for chemopreventive strategies to be applied for successful management as a prevention of progression strategy.^2^^,^^3^ More than half of all cancer patients report taking dietary supplements after they are told that they have cancer.^4^ Despite their popularity, there is a lack of clinical evidence that dietary supplements can reduce cancer progression.^5^ Thus, effective evidence-based treatments with limited side effects are urgently required.

Interest in the use of phytochemicals for the prevention or treatment of various cancers, including PCa, has increased considerably in recent years.^6^^,^^7^ Moreover, as with the standard of care therapies, the administration of phytochemical combinations offers considerable promise in improving outcomes. In fact, compared to treatment with a single agent, combination therapies provide several advantages, including better efficacy due to targeting/modulation of multiple cell signaling pathways, lower toxicity due to lower required doses, and potentially reducing the development of resistance to therapy.^8^

Several studies have evaluated the efficacy of phytochemicals and their combinations as preventive or therapeutic measures for PCa and other tumor types. We recently identified a combination of ursolic acid (UA) and curcumin (CURC) from a high-throughput screen of a natural product library with synergistic ATP depletion and reduction in cell survival in PCa cells, as well as synergistic inhibition of PCa tumor growth in vivo.^9^ CURC is a polyphenolic compound found in turmeric, a spice derived from *Curcuma longa* and UA is a pentacyclic triterpene compound found in various fruits and vegetables (e.g., apple peels). While there are no human trials combining UA with other compounds, combination therapy with CURC in other studies has been explored. For example, the combination of CURC and phenethyl isothiocyanate has been shown to inhibit PC-3 xenograft tumor growth to a greater extent compared to individual agents alone.^10^ The combination of CURC with soy isoflavones also significantly suppressed PSA production in a randomized double-blind controlled study in men undergoing prostate biopsies.^11^ Combinations of agents have also shown promise for increased activity at other cancer sites. For example, the combination of CURC and phospho-sulindac showed better inhibitory activity in a xenograft model using A549 cells than either agent alone.^12^

Despite promising pre-clinical results, there are major challenges in the translation of these results to human trials using natural products and dietary supplements, including good manufacturing practices (GMP), known active ingredients, bioavailability, and clinical trial rigor.^4^ In the current study, we addressed these challenges using a combination of UA + CURC in an academic run, Phase I clinical trial, with known active ingredients and enhanced bioavailability using GMP protocols.

## 2 Materials and Methods

### 2.1 Chemicals, Reagents and materials

Pooled human liver microsomes, sodium dihydrogen phosphate, disodium hydrogen phosphate, and magnesium chloride hexahydrate were purchased from Sigma-Aldrich. CURC, UA, 18β-Glycyrrhetinic Acid (18- βGlc), curcumin-d_6_ (C-d6), and NADPH were purchased from Cayman Chemicals (Ann Arbor, Michigan, USA). LC-MS-grade methanol (MeOH), acetonitrile (ACN), HPLC-grade water, ACS-grade formic acid, and dimethyl sulfoxide (DMSO) were purchased from Fisher Scientific (Pittsburgh, PA, USA).

### 2.2 Dosing target selection

The natural products of UA and CURC have been previously tested at high doses for safety, but have not traditionally had high bioavailability.^13^ Herein, we were limited in capsule design by capsule size and the number of capsules that could reasonably be consumed in a day.

UA: There are no current prostate cancer clinical trials on UA. The extrapolated human equivalent dose for UA is 22 mg/kg/day.^14^ Several trials have tested the oral administration of ursolic acid for diabetes (150 mg daily after fasting, NCT02337933) and to improve muscle function (500 mg/day, NCT02401113). We initiated our dose at 300 mg daily, based on animal and other human trials.^15^, ^16^, ^17^

CURC: CURC has been studied in several clinical trials without side effects, with doses of up to 8000 mg/day. A current prostate clinical trial has been reported in *Clinicaltrials.gov* testing 1000 mg/day after prostatectomy to prevent cancer recurrence (NCT02064673) and another study with CURC (3000 mg/day) as an adjuvant to radiation therapy (NCT01917890). Based on these studies, we tested our lipid-based formulation at a dose of 1200 mg/day.

### 2.3 CURC and UA capsule manufacturing

The capsules were designed by the Southwest Research Institute (SwRI) using good manufacturing practice (GMP) techniques. After purchasing UA and CURC, we performed milling to reduce the particle size and added lipid excipients to formulate microspheres. The final product was loaded into cellulose capsules. Further details are provided in the supplemental methods section.

### 2.4 Study design

We performed a standard 3 × 3 phase 1 design based on the safety evaluation of UA and CURC tested individually and then in combination (Study Schema in Supplemental Fig. 1). We modified the 3 × 3 design to include any toxicities because we selected the maximum dose due to the reported safety of large doses of these compounds. We enrolled subjects for a 2-week study period and used paired samples for individualized comparisons and pooled results for group comparisons.

### 2.5 Population and recruitment

We recruited healthy men enrolled in the San Antonio Biomarkers of Risk (SABOR) study, a community-based prostate cancer screening cohort. The San Antonio Biomarkers of Risk (SABOR) study cohort was funded by the 10.13039/100000054National Cancer Institute Early Detection Research Network-sponsored Clinical and Epidemiologic Validation Center since its nascent enrollment in 2000 (IRB# HSC20000030H). A separate IRB was approved for the Phase 1 trial (IRB# HSC20190940H). The participants had no prior diagnosis of prostate cancer at the time of enrollment and had varied prostate cancer risk profiles. We used data from the local UT Health San Antonio Data Safety and Monitoring Board (DSMB).

### 2.6 Study outcomes

Safety was compared to evaluate the number, frequency, duration, and relationship of toxicity events to CURC and UA, as defined by the Common Terminology Criteria for Adverse Events (CTCAE) v. 4.03. The secondary outcomes included the measured plasma levels of UA, CURC, and selected metabolites. We also investigated the impact on the microbiome in a paired analysis for changes and the overall theme of imputed bacterial function.

### 2.7 Measurement of compounds in blood

The compounds and their metabolites were measured using a UHPLC-MS/MS system on a Hybrid quadrupole-Orbitrap mass spectrometer (Q Exactive, Thermo Scientific, Waltham, MA, USA) hyphenated with a Thermo Scientific Vanquish Flex ultrahigh-pressure liquid chromatography (UHPLC) system via an electrospray ionization source. For the parallel reaction monitoring (PRM) scan, the resolution, automatic gain control (AGC) target, and maximum injection time (IT) were set at 17500, 2e5, and 100 ms, respectively. The NCE values for each metabolite were individually set, and the details are provided in Supplemental Table 1. The All-Ion Fragmentation (AIF) scan consisted of a scan range of *m/z* 100–750 with an NCE of 10. The resolution, AGC target, and maximum IT for the AIF scan were set at 70000 ms, 3e6 ms, and 200 ms, respectively. More details regarding the preparation and calibration are provided in the Supplementary Methods section.

### 2.8 P-glycoprotein (p-gp) assay

P-glycoprotein (P-gp/MDR1) is one of the most clinically important transmembrane transporters in humans responsible for excretion of drug at the level of the gut.^18^ HepG2 cells were plated in a white-walled, clear-bottom 96-well plate. The experiment was performed according to the manufacturer's instructions (ab284553; Abcam). Multi-drug resistant 1 (MDR1) activity was detected using a lipophilic non-fluorescent P-gp substrate. The substrate readily diffuses through the plasma membrane, where cytosolic esterases hydrolyze the substrate into an active hydrophilic fluorophore. The active fluorophore cannot pass through the membrane and remains trapped in the cell. Fluorescence was detected with a plate reader after 30-min incubation with varying concentrations of CURC and UA, and verapamil was used as a positive control.

### 2.9 Human microsomal metabolism

Liver microsomes are subcellular fractions which contain drug metabolizing enzymes (e.g., cytocrhomes P450) and can be used to determine the in vitro intrinsic clearance of a compound.^19^ Stock solutions (1.00 mg/mL) of CURC, UA, and IS (including C-d6 and BA) were prepared individually by dissolving 1 mg of each compound in 1 mL DMSO. The respective stock solutions were further diluted using MeOH to prepare calibration curve standards and working solutions. The diluent was prepared by spiking the IS (C-d6 and BA) in 100% MeOH to obtain a concentration of 100 ng/mL. The solutions were stored at −20 °C before use. The metabolic interaction between UA and CURC was investigated by incubation of UA (final concentration of 4 μM) either alone or with different concentrations of curcumin (1:1, 1:2, or 1:4 concentration ratios) together with pooled HLM microsomes in phosphate buffer (pH 7.4) at 37 °C ^o^C) and NADPH for 40 min at 37 °C. Aliquots of 30 μL were collected at different time points, and the reaction was terminated by adding 120 μL of the diluent. Finally, the collected samples were vigorously vortexed, centrifuged for 10 min at 4 °C and 50 μL of the clear supernatant was transferred to LC-MS vials for analysis. UHPLC–MS analysis was performed using a Thermo Scientific Vanquish UHPLC system coupled with an Orbitrap IQ-X Tribrid mass spectrometer with an electrospray ionization (ESI) interface. UHPLC analysis was performed in reverse phase mode with an ACQUITY BEH C18 150 × 2.1 mm (1.7 μm, 130 Å) column (Waters, Milford, MA), using a gradient elution with an organic phase of 0.1% formic acid in acetonitrile and an aqueous phase of 0.1% formic acid in water at a flow rate of 0.3 mL/min. The gradient elution ranging from 5% B:0–1 min; 5–95% B:1–8 min; 95% B:8–12.5 min, 95-5% B:12.5–13.5 min, followed by 5% B:13.5–19 min for post equilibration. The H-ESI interface was operated in negative ion mode with interface ion transfer tube temperature 320 °C, vaporizing temperature 250 °C, sheath gas 45 Arb, auxiliary gas 10 Arb, sweep gas 2 Arb, and detector voltage 3.5 kV. Quantification was achieved using the targeted-MS2 mode of ions at 455.3522 *m/z* for UA and 469.3314 *m/z* for 18β-Glc (internal standard). Data processing was performed using the Thermo Scientific Freestyle software (1.8. SP2 version).

### 2.10 Microbiome methods

We obtained rectal swabs from participants at the baseline visit and after 2 weeks and isolated DNA using our previously published methods.^20^ Genomic DNA was used to amplification of V1–V2 variable region of the 16S rRNA genes with custom-designed primers (F27/R534 and V3–V4 variable regions of the 16S rRNA genes, following the Illumina 16S metagenomic library preparation guide.^21^ Final libraries were quantified, normalized, pooled together, and sequenced by paired-end sequencing (2 × 300 bp) using the Illumina Miseq platform, and raw paired-end 16S rRNA reads were merged into consensus fragments and confirmed. Primary taxonomic assignment was performed using closed-reference OTU selection against the GreenGenes database using default parameters. PICRUSt was applied to rarefied taxonomic profiles to infer the functional categories associated with taxonomic composition. Alpha- and beta-diversity analyses were performed using QIIME. Primary differential abundance analysis between baseline and 2-weeks per treatment employed the paired *t*-test with log-transformation of feature values, supplemented by the paired Mann-Whitney test and unpaired significance tests for group-level comparisons. All computational analyses were performed separately for the V1V2 and V3V4 amplicon regions.

### 2.11 Statistical analysis

Demographics between groups were compared using Fisher's exact test for categorical variables and Student's t-test for continuous variables. Laboratory safety values were analyzed using a paired *t*-test for differences, with significance set at p < 0.05. ANOVA was used to compare the plasma levels of UA, CURC, and CURC metabolites. Least significant difference (LSD) was used as the post-hoc analysis test to compare values between groups.

## 3 Results

### 3.1 Population

**Fig. 1:**
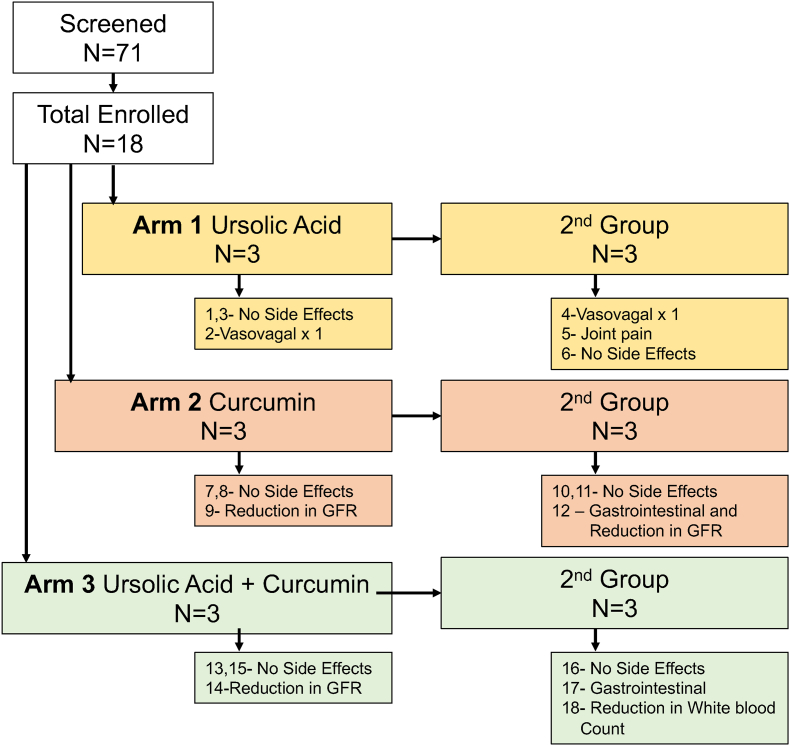
**Consort diagram.** Phase 1 design where each study group including curcumin, ursolic acid, and the combination are stand-alone 3 by 3 study designs. If any side-effects were identified, another 3 subjects were enrolled. The side effects for each study group are listed below the group name and sample size (n = 3). The arrow pointing left to right indicates that we enrolled a second group of 3 patients. If less than 3 subjects had side effects at the particular dose, then that dose was considered safe to proceed. In the case of natural products, very high doses such as several grams per day have been reported so the minimum tolerated dose (MTD) studies did not apply to this trial.

We contacted 71 subjects based on their active status and previous indications that they would be interested in subsequent studies (Consort Diagram, Fig. 1). Eighteen subjects were enrolled in a sequential trial. The demographics are displayed in Table 1. There were no statistically significant differences in the demographics between the groups. All 18 subjects completed the study, with zero dropouts due to side effects.

**Table 1:** **Demographics.** The initial papers leading to the experiments were based on the future treatment of prostate cancer; therefore, we enrolled healthy men to a phase 1 clinical trial with three cohorts that include ursolic acid (UA), curcumin (Curc) and the combination (CurcUA). In the first column we show the overall median and interquartile range (IQR) of demographics including some that are important during screening for prostate cancer. Then each of the 3 cohorts is displayed separately. The ANOVA test (continuous values) and the Fischer's Exact (categorical values) is used to compare the group for differences.

Baseline Demographics	Total Cohort (n = 18)	Ursolic Acid (n = 6)	Curcumin (n = 6)	CURC & UA (n = 6)	ANOVA
	Median (IQR) or Number (%)	Median (IQR) or Number (%)	Median (IQR) or Number (%)	Median (IQR) or Number (%)	P Value
Age	67 (60–72)	69 (59–75)	70 (64–74)	62 (60–67)	0.24
Race/Ethnicity					0.41
European/White	12 (67%)	4 (67%)	4 (67%)	4 (67%)	
Hispanic/Latino	3 (17%)	2 (33%)	0 (0%)	1 (17%)	
Black/African American	3 (17%)	0 (0%)	2 (33%)	1 (17%)	
Body Mass Index (BMI)	25.6 (24.2–31)	24.7 (24.0–27.4)	25.0 (23.1–32.8)	30.1 (25.2–31.5)	0.523
Active Smoker	2 (28%)	3 (50%)	0 (0%)	2 (33%)	0.14
Diabetes	3 (17%)	2 (33%)	1 (17%)	0 (0%)	0.47
Hypercholesterolemia	4 (22%)	2 (33%)	2 (33%)	0 (0%)	0.28
Hypertension	5 (28%)	3 (50%)	2 (33%)	0 (0%)	0.14
Family history of prostate cancer	5 (28%)	2 (33%)	2 (33%)	1 (17%)	0.76
Prostate Specific Antigen (PSA)	0.95 (0.73–1.38)	1.00 (0.38–2.23)	0.95 (0.73–1.48)	1.1 (0.68–1.38)	0.72
Abnormal prostate exam	0 (0%)	0 (0%)	0 (0%)	0 (0%)	0.99
Previous Negative Prostate Biopsy	2 (11%)	2 (33%)	0 (0%)	0 (0%)	0.11
Prostate Size on Exam					
Small (<30 gm)	12 (67%)	4 (67%)	4 (67%)	4 (67%)	0.99
Medium (30–60 gm)	6 (33%)	2 (33%)	2 (33%)	2 (33%)	0.99
Large (>60 gm)	0 (0%)	0 (0%)	0 (0%)	0 (0%)	0.99
Medications for Benign prostate hypertrophy (BPH)	0 (0%)	0 (0%)	1 (17%)	1 (17%)	0.57
Previous Antibiotics in the last 6 months	2 (11%)	2 (33%)	0 (0%)	0 (0%)	0.11
Previous vitamin or supplement use	14 (78%)	4 (67%)	5 (83%)	5 (83%)	0.73

### 3.2 Confirmation of capsule ingredients

The contents of the raw materials were 91.0% UA and 95.7% CURC. Further analyses were performed on the capsules after milling, lipid microsphere preparation, and addition of excipients. No alterations in the parent compounds were noted as 100% curcuminoids, and 100% UA structures were noted. The single capsule contained 41% CURC (200 mg) and 12.6% UA (50 mg). Neither capsule exhibited microbial contamination.

### *3.3* *Safety*: Overall, the supplements were well tolerated, and none of the patients stopped the trial due to side effects

- Common Terminology Criteria for Adverse Events (CTCAE) v 4.03. The CTCAE forms are presented in Table 2. A common low-grade subject complaint was gastrointestinal (GI)-related and was more attributed to ursolic acid (n = 3). There were two cases of neurological domain incidents. One vasovagal reaction occurred with the initial blood draw prior to drug ingestion and a presyncope episode in a man who donated blood earlier in the day, both in the CURC arm. We noted an increase in creatinine in three men who took UA, two in the UA alone group, and one in the combination group. An increase in creatinine levels indicates kidney dysfunction. These were laboratory values only, and no long-term effects or treatment were needed (grade 1).

**Table 2:** **Safety evaluation.** Subjects were evaluated at each study visit for adverse events and side effects using the standardized Common Terminology Criteria for Adverse Events (CTCAE) version 4.03. We have indicated the ID number, study group, if an event took place, the study grade, domain, and attribution. Only one grade 3 even took place and was attributed to blood draw and not attributed to the study drug.

ID Number	Group	Adverse Event	Event	Toxicity Grade (CTCAE v4.0)	Expected	Domain	Relationship To Study Treatment	Comments
CU-CD-01	Ursolic Acid	No						
CU-CD-02	Ursolic Acid	Yes	Vasovagal Reaction	2	N	Nervous system	Unlikely	Occurred prior to subjects first dose.
CU-CD-03	Ursolic Acid	No						
CU-CD-04	Ursolic Acid	Yes	Pre-syncope	2	N	Nervous system	Unlikely	Donated plasma earlier in the day.
CU-CD-05	Ursolic Acid	Yes	Joint pain	1	N	Musculoskeletal	Unlikely	Mild joint pain.
CU-CD-06	Ursolic Acid	No						
CU-CD-07	Curcumin	No						
CU-CD-08	Curcumin	No						
CU-CD-09	Curcumin	Yes	Creatinine increased	1	N	Blood/Urinary	Unlikely	Creatine increased from 1.10 to 1.24.
CU-CD-10	Curcumin	No						
CU-CD-11	Curcumin	No						
CU-CD-12	Curcumin	Yes	Diarrhea	1	Y	GI	Possible	
CU-CD-12	Curcumin	Yes	Bloating	2	Y	GI	Possible	
CU-CD-12	Curcumin	Yes	Flatulence	1	Y	GI	Possible	
CU-CD-12	Curcumin	Yes	Creatinine increased	1	N	Blood/Urinary	Unlikely	Creatine increased from 1.0 to 1.21.
CU-CD-13	Curc + UA	No						
CU-CD-14	Curc + UA	Yes	Creatinine increased	1	N	Blood/Urinary	Unlikely	Creatine increased from 1.13 to 1.32.
CU-CD-15	Curc + UA	No						
CU-CD-16	Curc + UA	No						
CU-CD-17	Curc + UA	Yes	Flatulence	1	Y	GI	Possible	
CU-CD-18	Curc + UA	Yes	White blood cell decreased	2	N	Blood/Urinary	Unlikely	Baseline low WBC count (3.1–2.4).

### 3.4 Laboratory safety values

Laboratory analysis was performed at baseline and at the end of the two-week study. We then subtracted the values (week 2: baseline) to obtain the differences for each participant. The summary values are displayed in Table 3, which shows the median difference and interquartile ranges (IQR) between the values.

o Renal function: Upon review of the laboratory values, we noted a concern regarding kidney function. Chronic kidney disease (CKD) is based on glomerulary filtration rate (GFR), and CKD stage 3 starts at a value below 60. One recipient enrolled in the CURC arm with a baseline GFR of 45, and after 2 weeks, the value was 46. Only one participant had a >60 GFR and dropped below that number. The participants also had the largest drop in GFR (17 points) from 76 to 59 in the combination (CurcUA) group. However, his plasma values for both CURC and UA were undetectable, and only a small amount of CURC glucuronide was present. Therefore, it is difficult to attribute poor kidney function to high UA level. Despite this, the creatinine rise and subsequent lowering of the GFR did not result in clinically meaningful side effects. However, kidney function should be closely monitored in future studies.
o Bicarbonate. Bicarbonate can be harmful if large changes occur in either direction, but clinicians are usually concerned about metabolic acidosis. We noted bicarbonate to be significantly different between baseline and two weeks in the combination group (CurcUA). In the case of the bicarbonate value in the study, they reduced the value by 2.5, making it less acidic. This situation may be beneficial to normal patients yet the value of 2.5 difference was of inconsequential clinical significance.
o Liver function. None of the patients had outward signs of liver complications, such as jaundice or pruritus. The alanine aminotransferase (ALT) were significantly different from baseline. Concerning liver functions will rise with time; however, we noted a reduction in ALT by 3.5 in the combination arm (CurcUA). The value would suggest improvement rather than harm, yet it is still of inconsequential clinical significance.

**Table 3:** **Safety laboratory results.** We display the differences between the week 2 values minus the baseline values. A minus sign will show a reduction in that value over 2 weeks. The p values were calculated using a paired T test for difference, where P < 0.05 is a significant difference. While there was a significant reduction in potassium in the curcumin cohort this was not clinically significant. In the CurcUA group, the statistically significant differences in bicarbonate, creatinine, glomerular filtration rate (GFR), and alanine aminotransferase (ALT) were not clinically significant changes.

Difference in Safety Laboratory Tests	Ursolic Acid (n = 6)	Curcumin (n = 6)	Ursolic Acid + Curcumin	UA (P value)	Curc (P value)	CurcUA (P value)
White blood cell (WBC)	0.20 (−0.23 to 0.85)	0.15 (−0.43 to 0.68)	0.15 (−0.73 to 0.38)	0.25	0.52	0.53
Hemoglobin	0.20 (−0.33 to 0.58)	0.20 (−0.30 to 0.55)	0.05 (−0.55 to 0.42)	0.66	0.37	0.76
Hematocrit	1.55 (−0.85 to 2.85)	0.25 (−1.45 to 2.35)	0.35 (−2.18 to 1.53)	0.39	0.74	0.71
Platelets	9.5 (2.5–27)	7.0 (−5 to 31.5)	0.0 (−7.5 to 8.0)	0.08	0.28	0.956
Sodium	0.5 (−1.5 to 2.25)	0.0 (−1.5 to 1.0)	0.05 (−0.8 to 1.3)	0.72	0.61	0.82
Potassium	0.15 (−0.08 to 0.45)	−0.15 (−0.3 to −0.8)	−0.05 (−0.3 to 0.2)	0.1	0.02*	0.9
Chloride	−1.0 (−2.75 to 1.75)	−0.5 (−1.3 to 0.8)	0.5 (−1.5 to 2.0)	0.85	0.82	0.84
Bicarbonate	1.0 (−1.5 to 3.0)	−1.5 (−4.3 to 0.0)	−2.5 (−3.3 to −0.5)	0.48	0.21	0.04*
Creatinine	−0.08 (−0.33 to 0.12)	0.07 (−0.07 to 0.16)	0.06 (0.03–0.98)	0.18	0.38	0.04*
Glomerular Filtration Rate (GFR)	−3.5 (−10.3 to 3.5)	−5.5 (−12.0 to 4.5)	−4 (−7.3 to −1.8)	0.36	0.54	0.03*
Calcium	0.03 (−0.05 to 0.48)	0.2 (−0.15 to 0.32)	0.1 (−0.23 to 0.32)	0.14	0.6	0.59
Bilirubin	0.0 (−0.08 to 0.15)	−0.1 (−0.3 to 0.0)	0.05 (−0.13 to 0.23)	0.84	0.06	0.56
Albumin	0.0 (−0.1 to 0.2)	0.05 (−0.05 to 0.2)	0.0 (−0.13 to 0.2)	0.61	0.46	0.81
AST	2.0 (−1.3 to 3.3)	2.0 (−2.0 to 3.3)	−1.0 (−2.3 to 0.8)	0.25	0.08	0.81
ALT	1.5 (1.0–12.8)	−1.0 (−3.25 to 0.0)	−3.5 (−5.0 to −1.5)	0.16	0.54	0.01*
Alkaline Phosphatase	2.5 (−2.5 to 5.0)	−2.0 (−5.5 to 9.3)	0.5 (−5.5 to 9.5)	0.35	0.81	0.56

• P value < 0.05.

### 3.5 Peak plasma levels (C_max_)

**Fig. 2:**
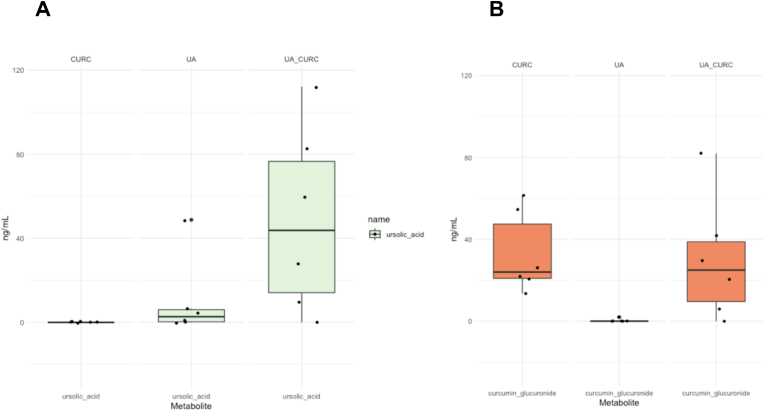
Plasma levels of UA and CURC glucuronide. A. Box plots containing the plasma levels of ursolic acid. There was a statistically significant difference between median levels of 2.7 ng/mL to 43.8 ng/mL from UA alone and the combination treatment group, respectively (P = 0.03). B. Box plots containing the plasma levels of CURC glucuronide. There was no statistical difference between the plasma levels of curcumin and the combination group (p > 0.05).

**Fig. 3:**
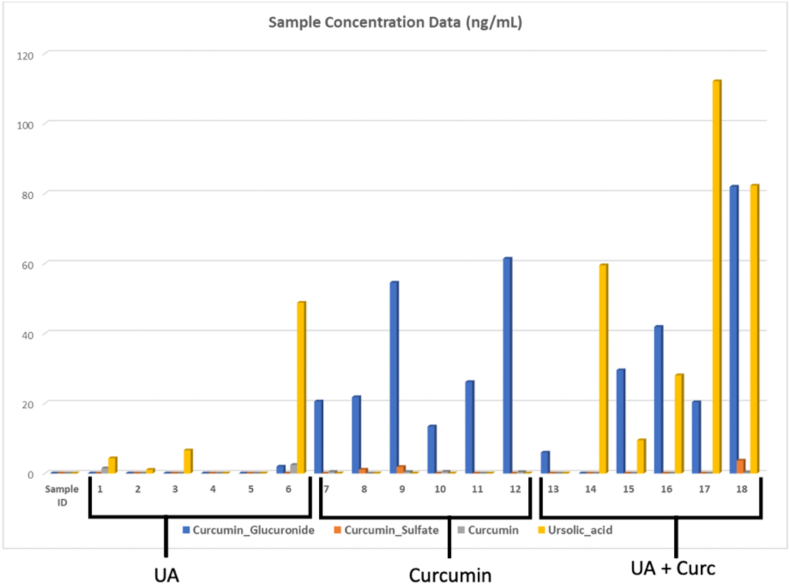
Plasma levels of natural compounds and metabolites. Bar graphs of the plasma levels of UA, CURC, and CURC metabolites (CURC sulfate and glucuronide). Subjects 1–6 received UA 300 mg/day with modest absorption of and only one subject with significant levels over 40 mg. The next 6 subjects (subjects 7–12) took 1200 mg/day of curcumin. We noted minimal amounts of the parent compound and large amounts of CURC glucuronide indicating. The last 6 subjects (13–18) were in the combination group (CurcUA) which as shown had statistically significant increases in UA and CURC glucuronide plasma levels.

The maximum concentration on day 14 was compared across the groups. In the UA alone cohort (Group 1: UA), the UA plasma values reached a median of 2.7 ng/mL (IQR 0.0–17.2 ng/mL) and were not statistically different from those in the CURC alone group that did not ingest UA (p = 0.5). The median plasma values of CURC and its metabolites in this group were 0.0 ng/mL as expected (Fig. 2). In the group assigned to CURC alone (group 2) the median plasma level of the parent compound was only 0.42 ng/mL (IQR 0.0–0.48 ng/mL). The highest levels of CURC were 0.52 ng/mL. The major metabolite detected was CURC glucuronide, with a median level of 24.0 ng/mL (IQR 18.8–56.3 ng/mL). There were no significant differences in CURC (0.42 vs. 0.0 ng/mL, p = 0.1) or CURC glucuronide (24 ng/mL vs. 25 ng/mL, P = 0.8) plasma levels between the CURC alone group and the combination group (CurcUA). As expected, UA was not detected in the CURC-only treated group. In the combination group (Group 3: CurcUA), CURC levels were low and similar to those observed in both the UA and CURC only groups (Fig. 3). Notably, the plasma levels of UA increased 16-fold (from a median of 2.7 ng/mL 43.8 ng/mL) in the combination treatment group (P = 0.03). Supplemental Figs. 2–4 provide chromatograms of standards, MS data for standards, and calibration curves for standards.

### 3.6 Human microsomal metabolism

**Fig. 4:**
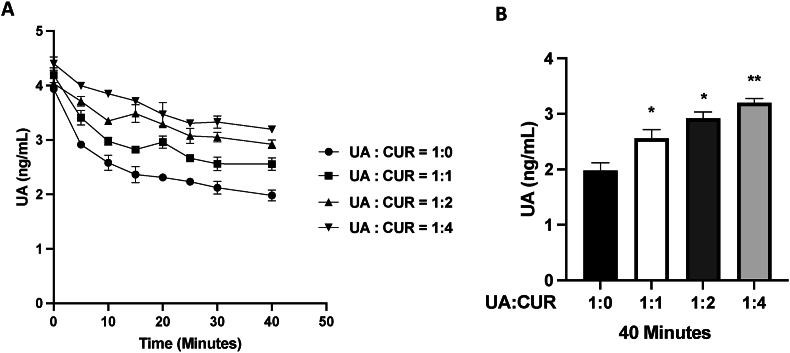
Liver microsome metabolism of UA in Combination with CURC. **A,** Time course of UA comsumption in liver microsomome with different concentration ratios of CURC added, including 1:0, 1:1, 1:2 and 1:4. The x axis represents the concentration of UA and a higher level represents less metabolism of the parent compound. The y axis demonstrates the time in minutes. **B,** Quantitation of UA present in the incubations at the end of the time course (40 min). *P < 0.05, **P < 0.01 One way ANOVA.

To further explore potential mechanisms for the altered plasma levels of UA when administered in combination with CURC, we examined the impact of different concentration ratios of CURC:UA in human microsomal incubations, as described in the Materials and Methods. Supplemental Fig. 5 shows the standard curve and LC-MS tracings for UA and the internal standard used for generating the data in Fig. 4. As shown in Fig. 4, increasing concentrations of CURC inhibited microsomal metabolism of UA. A ratio of 1:4 (UA: CURC), similar to the ratios of UA:CUR in the trial, significantly inhibited the metabolism of UA (73% remaining at 40 min compared with 47% remaining in UA-only incubations).

### 3.7 P-glycoprotein (p-gp)

CURC has been reported to inhibit P-gp^22^ therefore, we examined whether the combination of UA + CURC might further alter P-gp activity. For these experiments, HepG2 cells were treated with UA, CURC, or a combination of UA + CURC, and P-gp activity was measured as described in the Materials and Methods. Verapamil was used as the positive control to inhibit P-gp activity. As shown in Supplemental Fig. 6, CURC alone significantly inhibited P-gp activity (20 μΜ), whereas UA alone was significantly less effective at inhibiting P-gp activity. The combination produced various levels of P-gp inhibition, depending on the UA:CURC ratio.

### 3.8 Microbiome

Both V1–V2 and V3–V4 techniques were used to explore whether alpha diversity, as a measure of richness and evenness of gut bacteria, changed over the 2-week study period. Thus, the pre- and post-diversity values were compared using Simpson's Reciprocal Index and the Shannon index in box plots (Supplemental Fig. 7). The only significant difference was a reduction in alpha diversity in the V3–V4 Shannon index for CURC (p = 0.004). There was a trend (p = 0.06) in the combination group (CurcUA) toward increased bacterial diversity in V3–V4 Simpson reciprocal index. The beta diversity as a measure of similarity between groups showed significant differences using six different analysis techniques; all values were significant (p < 0.05, Supplemental Table 2). Supplemental Fig. 8 displays a principal coordinate plot (PCoA) to show that diversity was more associated with the individual person than the treatment groups assigned. Individually, the treatments changed the gut microbiome; however, we did not observe significant grouping over the short timeframe of the trial. Supplemental Fig. 9 provides a summary of the analysis to determine the specific bacteria and metabolic pathways affected by the groups. Bacterial associations included changes in dysbiotic bacteria (*Pseudomonas*,^23^ *Acidaminococcus*,^24^ *Tissierellaceae*,^24^ and *Mobiluncus*^25^ and healthy bacteria (*Roseburia*^26^ and *Porphyromonas*^27^). Bacterial production of biotin has been associated with a reduced risk of prostate cancer.^28^ Fatty acid metabolism is a target for prostate cancer prevention and treatment.^29^

### 3.9 Microbiome risk score

**Fig. 5:**
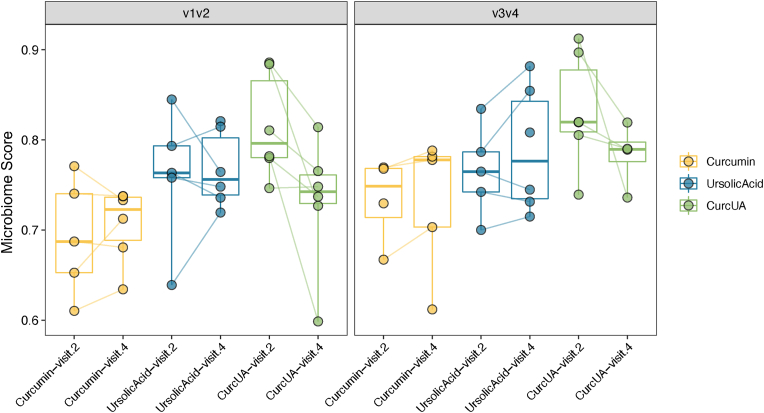
**Changes in the microbiome score for prostate cancer.** We display a side by side box and wisker plot to deomonstrate the calculated microbiome score using 10 metabolic pathways. On the left shows the original micorbiome score developed using the 16s V1V2 sequencing noting no changes from baseline to after intervertion in curcumin or ursolic acid. We do show signification reduction in the microbiome score in the combination group (paired T-test p = 0.044). On the right we show and imputed microbiome score using an 16s V3V4 data but is not statistically significant by paired T-test (all values p > 0.05).

Finally, we utilized our novel microbiome-derived risk score for prostate cancer based on 10 aberrant metabolic pathways (AUC = 0.64, p = 0.02) to determine the individual and combined effects on potential prostate cancer risk.^28^ There were no effects of CURC or UA individually. However, there was a statistically significant reduction in the microbiome score using the combination (paired T-test p = 0.044, Fig. 5). Although V3–V4 is not statistically significant by paired T-test, it trended in the same direction as the V1–V2 result. In order to provide a more rosbust score we combined regions V1–V2 and V3–V4 together and also achieved a significant difference (paired T-test, P = 0.006 for the CurcUA combination). In a multivariant general linear model (glm) adjusting for patient test group membership and 16S rRNA region, the glm indicated that there was a significant decrease in microbiome score from Visit 2 to Visit 4 independent of patient and 16s region (P = 0.0014).

## 4 Discussion

As noted in the introduction, the long latency of carcinogenesis makes PCa an ideal target for chemoprevention; however, to date, no approved chemopreventive agents have succeeded in the clinic. The current phase 1 clinical trial was designed to evaluate whether a combination of UA and CURC at the doses used would provide better bioavailability without significant toxicity. In addition, the trial was designed to examine the impact of short-term treatment with the combination on gut microbiome. Notably, combining these two phytochemicals in an oral dosage formulation resulted in a 16-fold increase in plasma UA levels (Fig. 2A). The levels of CURC (parent compound) were low in the plasma, regardless of whether it was administered alone or in combination with UA (Fig. 2B). CURC in the plasma was found primarily in its glucuronidated form. The finding of enhanced UA bioavailability given in combination with CURC is significant in that it represents the first evidence of orally dosed UA achieving measurable levels in the blood. Of potentially equal importance is the impact of oral dosing with phytochemicals on gut microbiome. The current data show that the combination of UA + CURC led to a sparing of bacteria with the capability of producing biotin. In addition, the combination of UA + CURC reduced a novel microbiome score predictive of prostate cancer risk. While the focus of the current study is prostate cancer, the current findings with this combination are likely to be of interest to other investigations using UA to target other cancers, obesity/diabetes, cardiovascular disease, brain disease, liver disease, and muscle wasting (sarcopenia).^30^

Regarding safety, the combination of UA and CURC was found to be safe and tolerable over a 2-week period. The overall safety was based on standardized criteria, and blood testing was used to measure systemic issues that may manifest later, particularly liver and renal function. Although there were minor changes in laboratory values, they were of little clinical significance, although renal, liver, and bicarbonate values will likely be evaluated in future clinical trials. Notably, a 21-patient phase 1 dose-escalation trial showed further safety using UA liposomes via intravenous administration over 14 days, and reported excellent tolerability in patients with advanced solid tumors.^31^ Important findings from this latter study included the finding of no accumulation of UA within the blood with multiple dosing suggesting safe clearance, and that IV administration obtained levels of nearly 1200 ng/mL without harm.

Regarding bioavailability, we found that CURC (the parent compound) was not readily available in the blood after oral ingestion using the current formulation and was largely present as curcumin glucuronide (Fig. 2B). This was somewhat expected, as previous studies have shown poor bioavailability of CURC.^32^ Furthermore, the presence of UA in the combination did not alter the blood levels of CURC glucuronide, which was similar to the levels observed in the CURC-only group. We did not test a non-lipid-enhanced version of CURC in this study due to previous bioavailability concerns. Notably, blood levels of UA were significantly higher when administered in combination with CURC than in the group receiving UA alone. UA is a lipophilic pentacyclic triterpenoid that undergoes P450-mediated metabolism, as well as phase II metabolic routes of glycine conjugation, glutathione conjugation, and glucuronidation.^33^^,^^34^ In particular, both UA and CURC are substrates of CYP3A4^35^^,^^36^ and inhibition of CYP3A4 has been shown to increase the bioavailability of UA.^36^ As shown in Fig. 4, human liver microsomal incubations with increasing concentration ratios of CURC:UA showed that CURC inhibited the metabolism of UA. These results suggest a possible mechanism for the increased blood UA levels when administered in combination with CURC. In this regard, CURC may have reduced P450 metabolism of UA in vivo, thereby increasing blood levels.

Another possible contributor to the altered blood levels is the ability of CURC to inhibit p-glycoprotein MDR1 active in the intestine, thereby increasing the blood levels of UA when administered in the combination. As shown in Supplemental Fig. 6, CURC inhibited MDR1 activity similar to the combination. Thus, it is possible that the inhibition of MDR1 contributed to increased blood levels of UA; however, more in-depth studies are needed to fully determine the contribution of this effect on overall blood levels.

Another important aspect of the present study was the analysis of the gut microbiome. Several studies have demonstrated an association between the gut microbiome and PCa.^37^ CURC beneficially alters the mouse microbiome and improves intestinal barrier function in the gut.^38^ In a randomized study of 14 subjects investigating the impact of CURC on human microbiome, it was found that CURC increased gut diversity by 67% compared to placebo, with a 15 % reduction in diversity.^39^ CURC has also been shown to reduce inflammation in mice and humans with ulcerative colitis (UC).^40^^,^^41^ UA has been shown to increase the abundance of beneficial bacteria, such as the phylum Firmicutes and genera Lactobacillus and Bifidobacterium, to prevent liver fibrosis.^42^^,^^43^ Additionally, UA is an anti-inflammatory agent that protects against the effects of UC, a disease that has been linked to a four-fold increased risk of prostate cancer in a case-control evaluation of 10,000 men in the United States.^44^^,^^45^ A second population-based study performed using the UK-biobank also noted a more modest but significant increase in the risk of prostate cancer in those with UC (n = 218,084, median follow-up 6.5 years, HR 1.47, 95% CI = 1.11–1.95, P = 00.007)^46^ and was confirmed in systematic reviews.^47^^,^^48^ To date, no studies have elucidated the effects of UA on the human gut microbiome.

In the current study, the combination of CURC and UA appeared to reduce the microbiome score (27) shown previously to be associated with prostate cancer risk (Fig. 5). The overall gut bacterial diversity was also improved by the combination more so than the individual compounds given alone. The biodiversity of all subjects was unique and did not cluster around treatment groups, but did show changes in composition individually. Using two types of variable regions (V1–V2 and V3–V4) allowed us to visualize consistency in the analysis. Beta diversity significantly changed in all groups, but microbiomes remained more closely related to the individual rather than to the interventional group (CURC, UA, CURC, and UA, all p < 0.01). Importantly, UA mitigated the loss of *Rosburia* caused by CURC alone and is associated with a healthy gut microbiome.^26^ Our previous publication on microbiome metabolic factors in prostate cancer concluded that men without cancer had more bacteria that produced biotin (vitamin B7) and this microbial ability was lacking in those men diagnosed with prostate cancer.^28^ Our current study suggests the combination of CURC and UA increased the microbes that have the ability to produce biotin and, as a secondary mechanism, may improve gut microbiomes in those at risk for prostate cancer.

While the current study provides some important findings for future trials, it has some limitations. First, we were unable to perform hourly pharmacokinetic measurements due to COVID restrictions at the time of the study. Second, the length of the study was short (2-weeks) and did not allow for an analysis of more long-term outcomes, especially on the microbiome. Third, the study was underpowered to extrapolate the microbiome findings to functional analysis due to the small population size. Even though the parent compound, CURC, did not reach measurable blood levels, CURC has been known to impact the gut microbiome and have associated metabolic effects that were not measured in this study.^49^ In addition, previous studies have suggested the β-glucuronidase is active in prostate tumor cells and may reconvert the CURC-glucuronide back to CURC at the tissue level, which could potentially impact tumor cells together with UA, especially if there was any tissue accumulaton of the compounds.^50^^,^^51^ The median level of UA in the group receiving the combination reached 43.8 ng/mL and it remains relatively unknown if this level would influence prostate cellular function. The small sample size in each group also limits the ability to perform subanalysis on subgroups known to have impacts on the microbiome such as Body Mass Indexes (overweight and obese), smoking habits, blood pressure levels, and metabolic conditions. Finally, alternative dosing regimens could not be evaluated because of the high cost of this study.

## 5 Conclusion

In conclusion, the current data provides evidence that combining UA and CURC in a lipid formulation can lead to increased blood levels of UA. Several mechanisms could potentially explain, at least in part, the observed increased blood levels of UA when administered in combination. In addition, some important information on the impact of the combination on gut microbiome was also obtained. Future studies with this combination will include a longer dosing period and substituting a more bioavailable form of CURC to improve its blood levels. The current results may also provide useful information for other potential uses of UA. Finally, the results of this study has provided valuable information for planning future clinical studies with this or related phytochemical combinations.

## Data access statement

Research data supporting this publication are available upon request to the authors.

## Funding: MAL

MAL is supported through the 10.13039/100014039DOD Prostate Cancer Research Program (10.13039/100014039PCRP) Physician Research Training Award. This work was supported by the Office of the Assistant Secretary of Defense for Health Affairs through the 10.13039/100014039Prostate Cancer Research Program under Award No. W81XWH-15-1-0441. Opinions, interpretations, conclusions, and recommendations are those of the authors and are not necessarily endorsed by the Department of Defense. MAL was additionally supported by the department of Urology, NCI CCSG grant (P30CA054174), and Roger L. and Laura D. Zeller Charitable Foundation Chair in Urologic Cancer. **JD and ST:** Research reported in this publication was also supported by the 10.13039/100000002National Institutes of Health under award number R01CA228404.

## Declaration of competing interest

The authors declare the following financial interests/personal relationships which may be considered as potential competing interests:

Michael Liss has patent #WO2023091668A1 issued to John DiGiovanni.
