# Supplementary material for: Phase 1 clinical trial evaluating safety, bioavailability, and gut microbiome with a combination of curcumin and ursolic acid in lipid enhanced capsules
Source: J Tradit Complement Med. 2024 Mar 7;14(5):558–67. doi: 10.1016/j.jtcme.2024.03.002 (PMC11384084; doi:10.1016/j.jtcme.2024.03.002)
Supplement: Multimedia component 1 [file mmc1.pdf]

Supplemental Table 1: The information of targeted analytes/metabolites

Analytes/IS	RT (min)	[M-H]-	Major Transition/Base Peak	NCE	Mode	LLOQ (ng/mL)	Linear Range (ng/mL)	Linear Regression Equation & R2	Calibration Type	Analyte used for Calibration	IS Used for Calibration
Curcumin	8.40	367.38	149.06	20	PRM	0.1	0.1-1000	Y = 0.00563874+0.00193131*X R^2 = 0.9983	Internal Calibration	Curcumin	Curcumin-d_6
Curcumin Sulfate	7.16	447.44	217.07	20	PRM	N/A	N/A	N/A	External Calibration	Curcumin Sulfate-d_6	Curcumin-d_6
Curcumin β-D-Glucuronide	6.61	543.50	113.02	15	PRM	N/A	N/A	N/A	External Calibration	Curcumin β-D-Glucuronide-d_3	Curcumin-d_6
Curcumin Sulfate-d_6	7.16	453.00	220.07	20	PRM	1	1-1000	Y = 0.000542484+0.000370986*X R^2 = 0.9968	External STD to quantify Curcumin Sulfate	N/A	N/A
Curcumin β-D-Glucuronide-d_3	6.61	546.50	113.02	15	PRM	0.5	0.5-1000	Y = 0.000943129+0.000203698*X R^2 = 0.9973	External STD to quantify Curcumin Glucuronide	N/A	N/A
Curcumin-d_6	8.40	373.42	152.08	20	PRM	N/A	N/A	N/A	IS	N/A	N/A
Betulinic acid	7.63	455.70	455.25	10	AIF	N/A	N/A	N/A	IS	N/A	N/A
Ursolic acid	11.77	455.70	455.35	10	AIF	1	1-1000	Y = 0.0235383+0.00196138*X R^2 = 0.9978	Internal Calibration	Ursolic acid	Betulinic acid

Supplemental Table 2. Microbiome Beta Diversity

	PERMANOVA evaluation of Tx Exposure Effect					
**Algorithm-distance-region**	**Df**	**SumsOfSqs**	**MeanSqs**	**F.Model**	**R2**	**Pr(>F)**	
liss-human-bray_curtis-v1v2	2	0.999	0.4995	4.6224	0.08965	0.0002	***
liss-human-bray_curtis-v3v4	2	0.7356	0.36782	3.5862	0.08354	0.0002	***
liss-human-unweighted_unifrac-v1v2	2	0.4292	0.21462	3.0867	0.0858	0.0002	***
liss-human-unweighted_unifrac-v3v4	2	0.3882	0.19412	2.3448	0.08745	0.0002	***
liss-human-weighted_unifrac-v1v2	2	1.4002	0.70008	3.7461	0.08983	0.0002	***
liss-human-weighted_unifrac-v3v4	2	0.13724	0.06862	2.6492	0.08981	0.02	*
insight-human-bray_curtis-v1v2	2	0.7496	0.37482	3.4679	0.08144	0.0002	***
insight-human-bray_curtis-v3v4	2	0.6383	0.31916	3.2705	0.0851	0.0002	***
insight-human-unweighted_unifrac-v1v2	2	0.5267	0.26336	2.1036	0.07778	0.0002	***
insight-human-unweighted_unifrac-v3v4	2	0.6562	0.32812	1.6093	0.08196	0.0002	***
insight-human-weighted_unifrac-v1v2	2	2.149	1.07449	3.6299	0.0927	0.0002	***
insight-human-weighted_unifrac-v3v4	2	0.12739	0.06369	2.3751	0.07198	0.0114	*

Supplemental table 2. The table shows the various statistical comparisons used to compare the beta diversity noting a statistically significant difference using several different computational comparisons. After adjusting for patient-to-patient variation and timepoint, gut microbiota composition is associated with at least 1 treatment exposure.

Supplemental Figure 1: Study Design

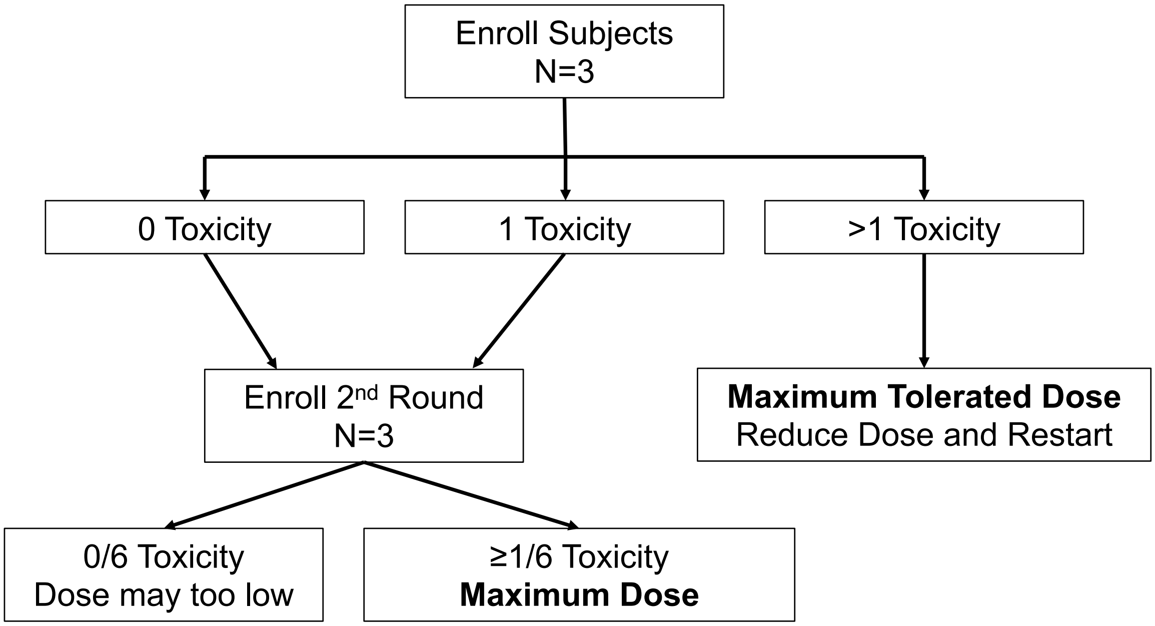

Supplemental Figure 1: Study Schema. We show a study schema displaying the 3 by 3 study design for a phase 1 clinical trial. Given the study of natural products with established safety profiles we included laboratory values and any side effects in the toxicity profiles.

Supplemental Figure 2: Extracted ion chromatogram of Standard and IS

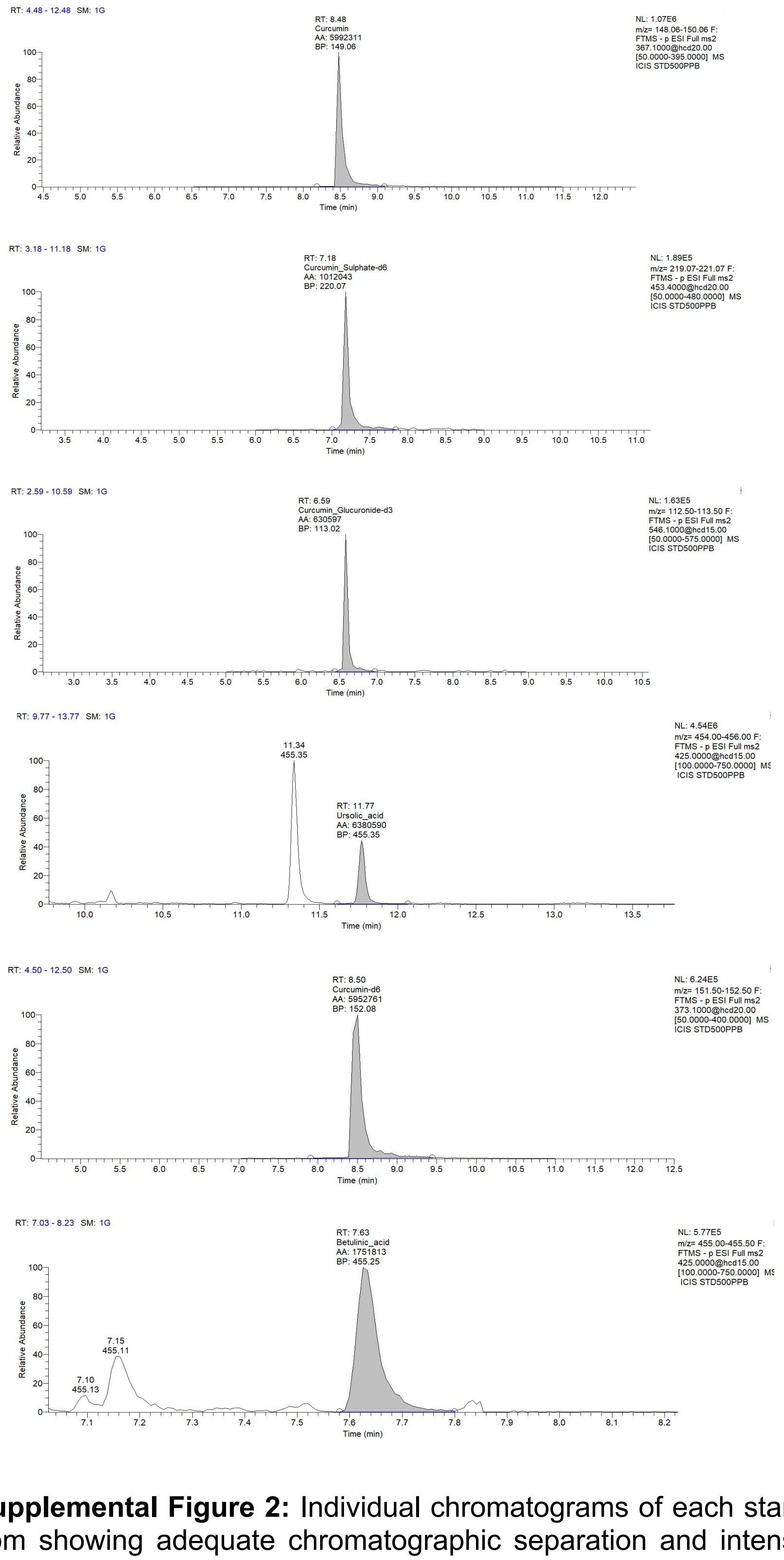

Supplemental Figure 2: Individual chromatograms of each standard at a concentration of 500 ppm showing adequate chromatographic separation and intensity achieved with the selected method.

Supplemental Figure 3: MS/MS spectra of Standards/IS

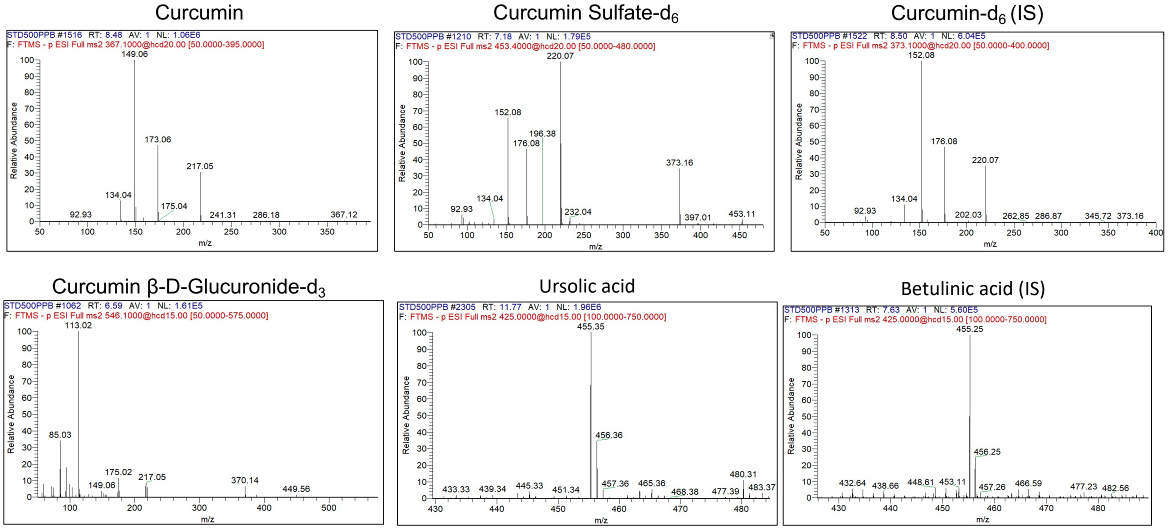

Supplemental Figure 3: MS2 spectrum for each standard at a concentration of 500 ppm showing the fragmentation patterns of each compound detected following application of the respective normalized collision energies (NCE) (See Table S1).

Supplemental Figure 4: Calibration plot of Standards

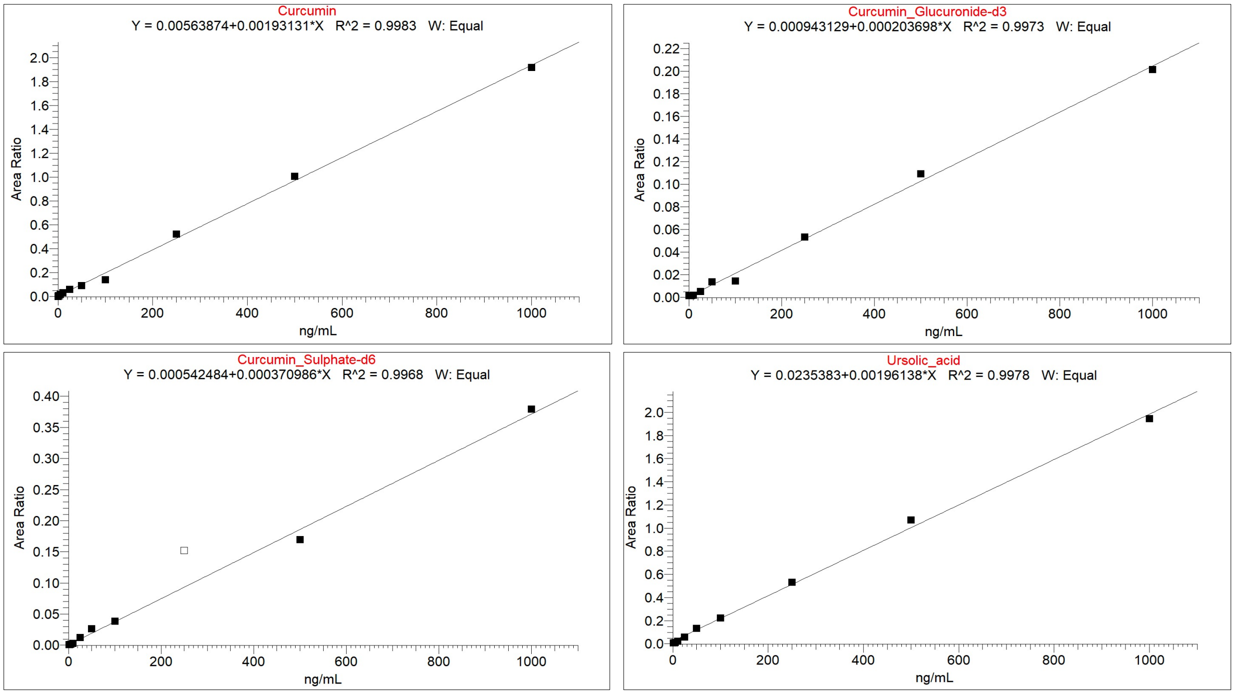

Supplemental Figure 4: Calibration curves for each standard showing adequate sensitivity and optimum linear range. These curves were used to create the regression equations to calculate the absolute concentrations of each related compound in the human plasma samples.

Supplemental Figure 5. Calibration of UA measurements in liver microsome studies.

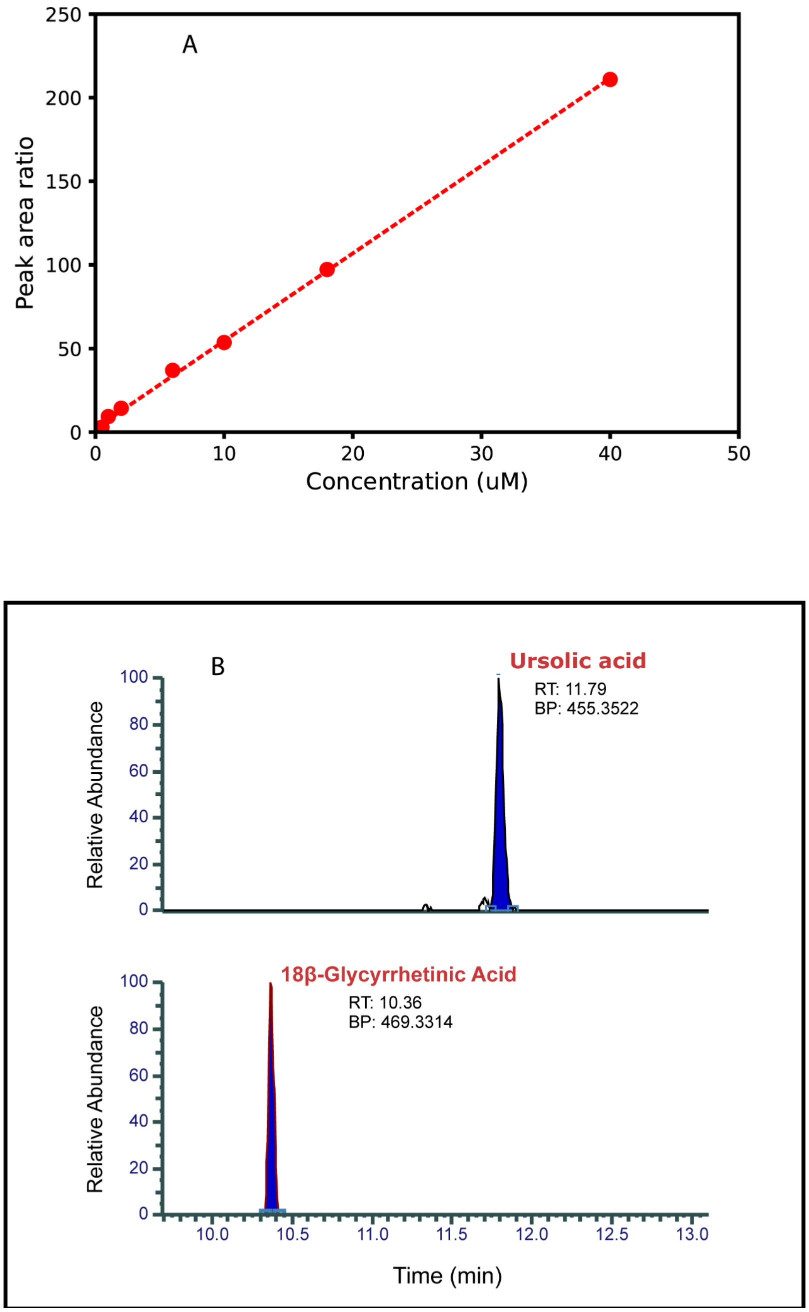

Supplemental Figure 5. A. Calibration curve of different UA concentrations against the peak area ratio of UA to its internal standard. **B.** Base peak chromatographic separation showing resolution of 1 μM UA and 100 nM 18β-Glycyrrhetinic Acid.

Supplemental Figure 6

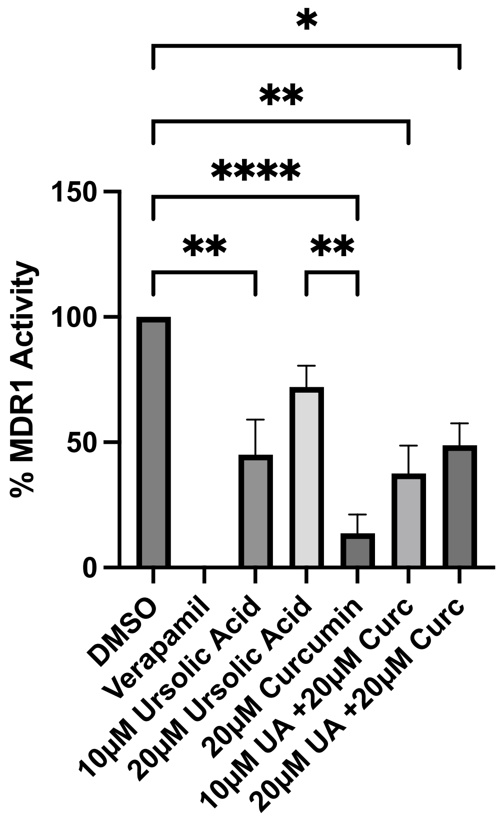

Supplemental Figure 6: Multi-Drug Resistance 1 (MDR1) Activity After Treatment with CURC and UA. % MDR1 activity of HepG2 cells using a bar chart. MDR1 activity was evaluated following 30-minute treatment with varying concentrations of curcumin and ursolic acid. Data is represented as an average of three experiments. Statistical analysis was performed using a one-way ANOVA with Tukey's multiple comparisons test and significance was set at $p < 0.05$. ($p < 0.05$ = *, $p < 0.01$ = **, $p < 0.0001$ = ****)

Supplemental Figure 7. Microbiome Alpha Diversity.

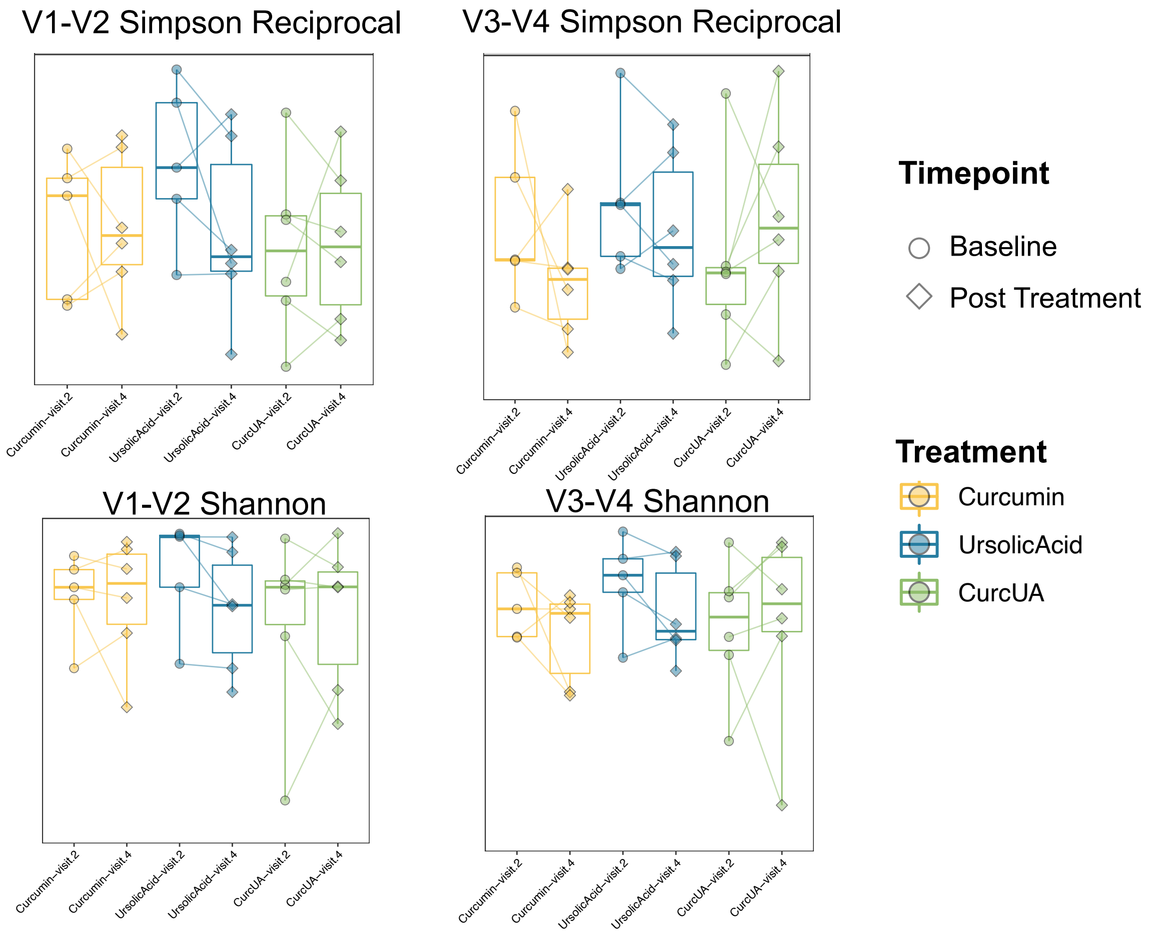

Supplemental Figure 7. Microbiome Alpha Diversity. We display four separate box plots noting alpha diversity, which measures the diversity of species. The top two box plots represent the Simpson's reciprocal index which quantifies biodiversity by considering richness and evenness of the microbiome representing overall biodiversity. Richness represents the number of species and evenness represents the proportional abundance of those species. We use two sets of variable regions (V1-V2 and V3-V4) to insure robustness in the analysis. The Shannon index is a commonly used and considered a standard diversity measure by dividing the number species in a group by the total number of individuals in the community. The box plots represent these values as pairs (visit.2=baseline represented by circles and visit.4=end of study, represented by diamonds). The circles (baseline) are connected to the end of the 2 weeks study levels (diamonds) by a line. The box represents the 95% confidence interval and line represents the median. In general, we note that curcumin and ursolic acid may lower overall alpha diversity, whereas the combination treatment improves overall diversity. Using the paired t-test, we compared the difference of pre and post intervention. All V1-V2 values (Shannon or Simpson reciprocal) showed not statistical differences (all $p>0.05$). We did note statistical differences in V3-V4 curcumin reduction in alpha diversity ($p=0.004$) and a trend for improvement of alpha diversity for the combination (CurcUA, $p=0.06$).

Supplemental Figure 8. Microbiome Beta Diversity.

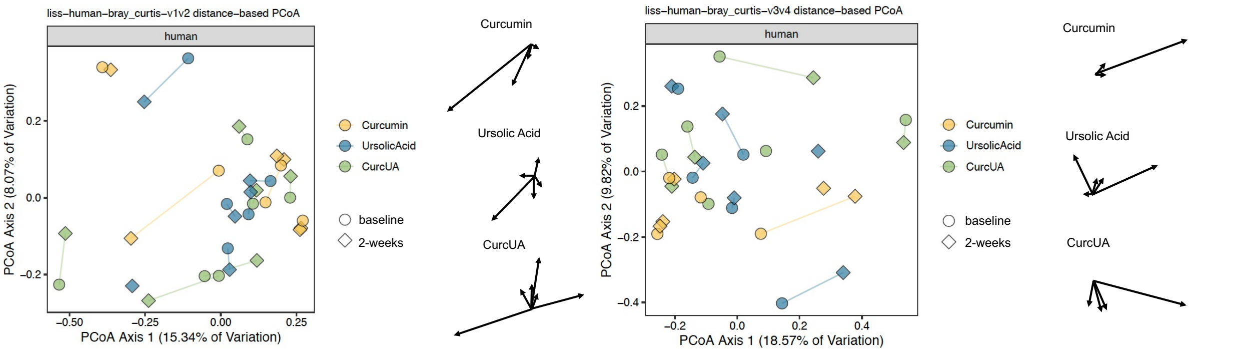

Supplemental Figure 8. Microbiome Beta Diversity. The top figures represent the principal coordinate analysis (PCoA) using Bray-Curtis calculated distances that represent similarity between groups (left V1-V2 and right V3-V4). We compare the change before and after treatment of curcumin (yellow), ursolic acid (blue), or the combination (green). The circle represents the baseline composition, and the diamond represents the gut microbiome composition after treatment. To the right of each PCoA plot, we have summarized the direction and distance from a fixed point to compare the differences in subjects. The direction does not represent a good or bad result, only different composition. Curcumin may be driving the beta diversity because in both curcumin and the combination (CurcUA) seem to have a directional component to changing diversity though they seem to be in opposite directions.

Supplemental Figure 9. Gut microbiome changes

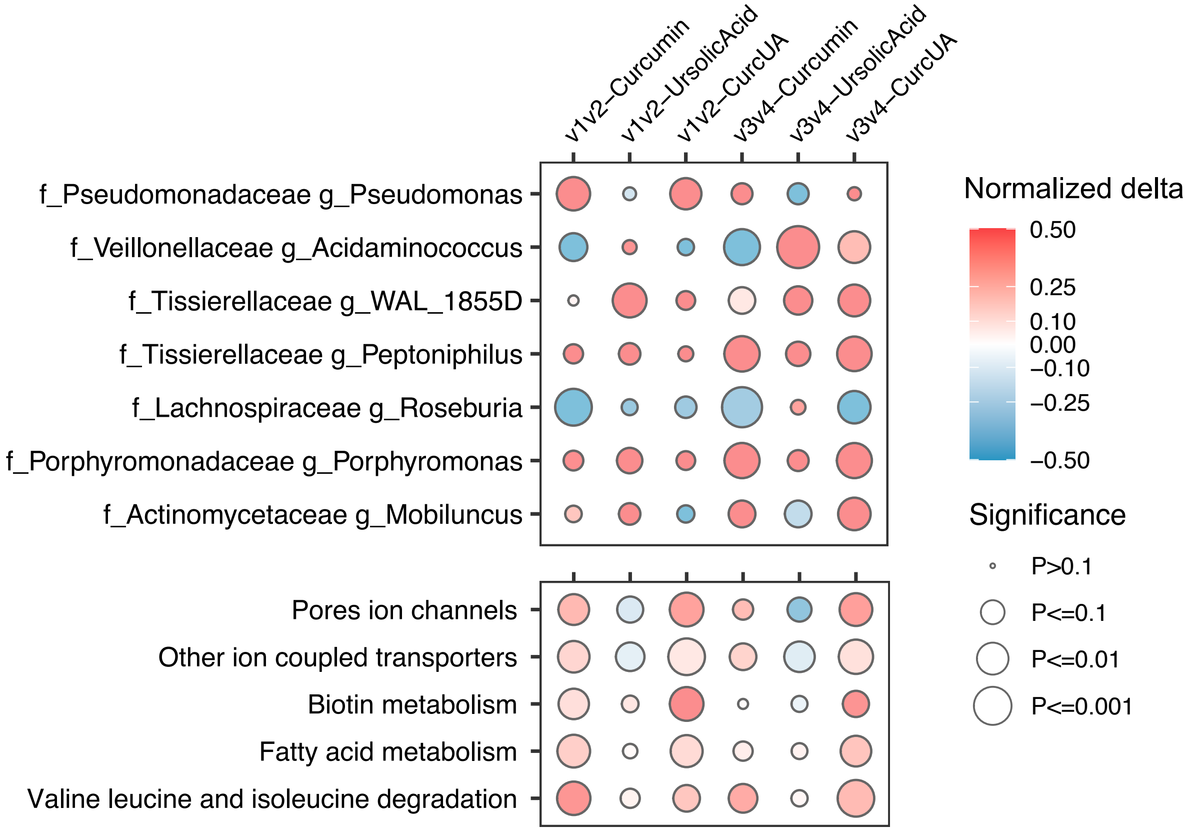

Supplemental figure 9. Gut microbiome changes. We display the changes in the gut microbiome over a 2-week period. At the top of the graph, the study group (curcumin, ursolic acid, or combination) is displayed along with the variable regain (V1-2 or V3-4) that was sequenced. The variable regions can provide different results therefore we display all the data for visual comparison, in that, if consistent the result is more robust. The figure legend on the right shows the change (delta) in the value over the two weeks after normalization. The circles correspond to specific p-values. The top box shows specific taxa of bacteria that change over two weeks in each group. The bottom box shows the metabolic pathways that change over time.
